# Primary Total Knee Arthroplasty for Treating Osteoarthritic Knees with Neglected Patellar Dislocation

**DOI:** 10.3390/medicina60091492

**Published:** 2024-09-13

**Authors:** Sung Eun Kim, Seong Hwan Kim, Jung-In Lee, Hyuk-Soo Han, Myung Chul Lee, Du Hyun Ro

**Affiliations:** 1 Department of Orthopedic Surgery, Seoul National University Hospital, Seoul 03080, Republic of Korea; paxgospel@gmail.com (S.E.K.); haha940120@gmail.com (J.-I.L.);; 2 Department of Orthopedic Surgery, Chung-Ang University Hospital, Seoul 06973, Republic of Korea; ksh170177@nate.com

**Keywords:** total knee arthroplasty, osteoarthritis, neglected patellar dislocation, outcomes

## Abstract

*Background and Objectives*: Neglected patellar dislocation in the presence of end-stage osteoarthritis (OA) is a rare condition characterized by the patella remaining laterally dislocated without reduction. Due to the scarcity of reported cases, the optimal management approach is still uncertain. However, primary total knee arthroplasty (TKA) can serve as an effective treatment option. This study aimed to present the clinical and radiological outcomes achieved using our surgical technique. *Materials and Methods*: A retrospective review of 12 knees in 8 patients with neglected patellar dislocation and end-stage OA who underwent primary TKA was conducted. The surgical procedure involved conventional TKA techniques (e.g., medial parapatellar arthrotomy) and additional procedures specific to the individual pathologies of neglected patellar dislocation (e.g., lateral release, medial plication, and quadriceps lengthening). Clinical outcomes, including patient-reported outcome measures (PROMs) (Knee Society Scores and the Western Ontario and McMaster Universities Osteoarthritis Index) and knee range of motion (ROM), were assessed preoperatively and two years postoperatively. Radiological measures including mechanical femorotibial angle and patellar tilt angle were assessed preoperatively and until the last follow-up examinations. Any complications were also reviewed. *Results*: There were significant improvements in all PROMs, knee ROM, and radiological outcomes, including mechanical femorotibial angle and patellar tilt angle (all *p* < 0.05). At a mean follow-up of 68 months, no major complications requiring revision surgery, including patellar dislocation, were reported. *Conclusions*: Primary TKA is an effective procedure for correcting various pathologies associated with neglected patellar dislocation in end-stage OA without necessitating additional bony procedures. Satisfactory clinical and radiological outcomes can be expected using pathology-specific procedures.

## 1. Introduction

Total knee arthroplasty (TKA) is an effective method for treating end-stage knee osteoarthritis (OA). TKA has shown excellent results and low incidence of complications at long-term follow-up [1]. However, anatomical pathologies combined with OA can negatively affect the outcomes of TKA. When surgeons encounter significant deformities of the knee, the likelihood of TKA complications increases, which can significantly affect the overall outcome [2,3,4].

Neglected dislocation of patella is a rare condition characterized by the unreduced, lateral displacement of the patella [5]. Whether the condition is congenital or secondary to trauma, it induces an increased valgus vector on the knee and external torsion on the tibia. Such alterations lead to permanent patellar dislocation, progressive deformities, and subsequent development of OA [6,7]. Valgus malalignment, lateral dislocation of the patella, and knee extensor weakness in addition to OA are commonly associated with this condition [8]. In adults, this condition tends to be diagnosed late due to the delayed onset of symptoms following secondary osteoarthritis. Chronic unreduced dislocation may go unnoticed because of patients’ functional adaptation, leading to a delay in diagnosis, which is often only made in the context of degenerative changes in the tibiofemoral joint [9].

The optimal management strategies and prognoses for these patients remain unclear. Prior studies on this topic, mostly case reports with fewer than three patients, have been inconsistent regarding surgical techniques and clinical outcomes [6,7,8,10,11,12,13,14,15,16,17,18]. Due to the complex deformities associated with neglected patellar dislocation, surgeons may encounter challenges in determining the appropriate surgical approach, compounded by the scarcity of literature on the subject. Thus, the establishment of a more standardized treatment protocol is required. We hypothesized that TKA may serve as a useful treatment for patients with these conditions to restore a normal lower limb alignment and improve functionality.

In this study, we present clinical outcomes of 12 cases involving neglected patellar dislocation accompanied by OA, all of which underwent primary TKA at our institute. Therefore, the purpose of this case series was to present the clinical and radiological outcomes following the management of neglected patellar dislocation through TKA. Furthermore, this series addressed the additional surgical procedures within the scope of TKA and necessitated by the specific pathologies that can be encountered during surgery. 

## 2. Methods

This retrospective study was approved by the Institutional Review Board of the authors’ institute (IRB No. 1808-094-966). From April 2014 to November 2018, a total of 13 knees of 9 patients with OA and neglected patellar dislocation underwent TKA. One patient was excluded due to a follow-up period of less than 1 year after the index surgery. Consequently, medical records of the remaining 12 knees from 8 patients were reviewed. Patient demographics and preoperative data are presented in Table 1. The mean age at the time of TKA was 67.6 years (range, 57 to 76), and there were 6 females and 2 males. The mean follow-up period was 68 months (range, 41 to 92). 

### 2.1. Radiographic Evaluation

**Figure 1:**
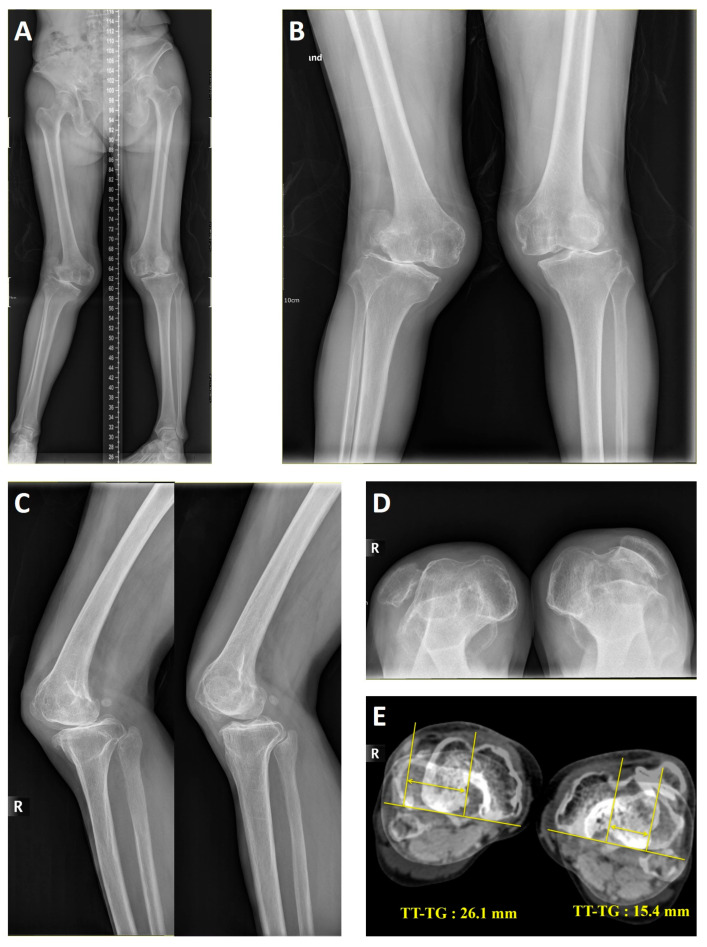
(**A**–**E**). Bilateral secondary osteoarthritis with neglected dislocation of the patella in a 68-year-old female patient (Cases No. 1 and No. 2): (**A**) long-leg standing anteroposterior view; (**B**) standing anteroposterior view; (**C**) lateral view of right and left knees; (**D**) skyline view; and (**E**) rotational computed tomography image showing the tibial tuberosity–trochlear groove (TT-TG) distance.

Parameters such as the mechanical femorotibial angle (MFTA), tibial subluxation, tibial external torsion, tibial tuberosity lateralization, trochlear dysplasia, tibial tuberosity–trochlear groove distance (TT-TG), and patellar hypoplasia were determined from preoperative radiographs and CT scans (Figure 1). These radiographs included the long-leg standing anteroposterior (AP), knee standing AP, knee 30° flexion lateral, and axial (skyline) views. The MFTA was defined as the angle formed between the line connecting the hip and knee centers and the line connecting the knee and ankle centers. Tibial subluxation on the femur was defined as a mismatch of over 5mm between the medial or lateral tibial and femoral borders in the coronal plane. The external tibial torsion was assessed, using CT scans, by measuring the angle between the tibial axis from the proximal tibia and the bimalleolar axis at the distal tibia [19]. A TT-TG over 20mm was defined as tibial tuberosity lateralization [20]. Trochlear dysplasia followed the classification of Dejour [21], while patellar hypoplasia denoted a flattened articular surface affixed to the lateral knee [22]. 

### 2.2. Surgical Technique

**Figure 2:**
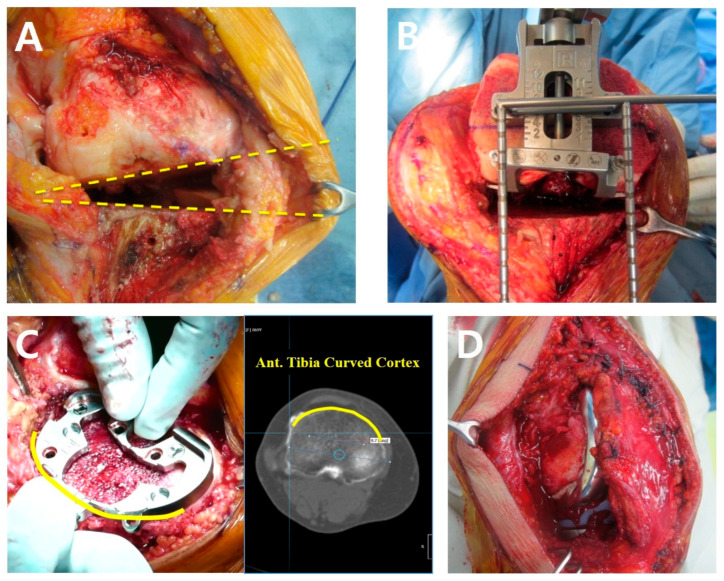
(**A**–**D**). Intraoperative photographs. (**A**) Medial structures stretched due to valgus deformity. (**B**) Achieving a rectangular flexion gap by holding the leg in a 90° knee flexion position with gravity gap tension. (**C**) Use of the anterior tibial curved cortex (ATCC) rotational alignment technique for tibial-component positioning. (**D**) Lateral structures left open post-release while the medial capsule is repaired.

All surgical procedures were performed using a conventional technique (Figure 2). After an anterior midline skin incision, a medial parapatellar arthrotomy was performed. The meniscotibial part of the deep medial collateral ligament (MCL) was released as a preliminary step, which was performed minimally because of the stretched medial structures due to valgus deformity. Both cruciate ligaments were resected in all cases. The choice of implants was based on the surgeon’s preference at the time of surgery, including the following systems: Triathlon (Stryker, Mahwah, NJ, USA), LPS, LCCK, Persona (Zimmer Biomet, Warsaw, IN, USA), and Attune (DePuy Synthes, Warsaw, IN, USA). An intramedullary guide was used for distal femur resection, while an extramedullary guide was used for proximal tibia resection. The bone cutting was made perpendicular to the mechanical axis of the femur and the tibia and planned from long-leg films taken preoperatively. 

After bone cutting, the extension gap and the alignment were checked using a tensor device. When the lateral extension gap was narrowed due to valgus deformity, a transverse stab incision and the pie-crusting technique were applied to the iliotibial band (ITB), posterolateral corner (PLC), lateral collateral ligament (LCL), and popliteus tendon to restore the rectangular gap balance. Femoral component rotation was determined using the gap technique, which involved creating a rectangular flexion gap while holding the leg in a 90° knee flexion position with gravity gap tension, using the weight of the patient’s lower leg [23]. After the anteroposterior cut was made using a femoral cutting guide, the flexion gap was measured. 

The anterior tibial curved cortex (ATCC) rotational alignment technique was used to determine tibial-component rotation [24]. Specifically, after the proximal tibia was cut, the anterior surface of the tibial baseplate was aligned with the ATCC of the proximal tibia. If lateralization of the tibial tuberosity was observed, the tibial component was correspondingly lateralized to account for this deviation.

Patellar thickness was measured using a caliper. Only patellae with an International Cartilage Repair Society Grade (ICRS) of over 2 were chosen for resurfacing. Of these, patellae with a thickness greater than 12mm at their most prominent dimension were resurfaced to minimize the risk of patellar fracture [25]. Among them, ten patellae—including all seven that were hypoplastic—were resurfaced using an all-polyethylene component. Cement was applied to augment any thin areas.

After all prostheses were fixed with cement, extensive lateral release of synovium, lateral retinaculum, and lateral patellofemoral ligament was performed in all knees. Medial plication was performed in two cases to maintain central patellar tracking, whereby the medial retinaculum was advanced to the patellar medial border. In two cases, a quadriceps snip was performed to improve surgical visualization. In another case, a V-Y quadricepsplasty was conducted to address the shortened quadriceps tendon by lengthening both the vastus medialis and vastus lateralis. This involved making an inverted V-shaped incision along both the lateral and medial portions of the quadriceps tendon, which was then repaired to achieve the appropriate tension (Table 1). For this case, a condylar-constrained knee prosthesis (LCCK) was used. Before closure of the wound, an intraarticular drain was inserted.

### 2.3. Postoperative Protocol 

All patients followed a standardized rehabilitation protocol, which included walking in the ward on the day of surgery with the assistance of a walking aid while wearing a knee immobilizer. On the second day, the intraarticular drain was removed, and continuous passive motion exercises were initiated. Patients were also encouraged to engage in quadriceps strengthening and straight-leg raise exercises.

### 2.4. Outcome Assessments

All patients underwent clinical and radiological assessments prior to surgery. Follow-up visits were scheduled for four weeks, three months, and twelve months after surgery, and annually thereafter. Patient-reported outcome measures (PROMs), including the Knee Society Score (KSS) and the Western Ontario and McMaster Universities Osteoarthritis Index (WOMAC), were collected preoperatively and two years postoperatively. Knee ROM data, such as knee flexion contracture and flexion angle, were also collected. Serial radiological assessments, along with clinical assessments, were performed at each visit to monitor for complications such as infection, instability, dislocation, or implant malposition. Radiological indices, specifically the mechanical femorotibial angle and the patellar lateral tilt angle, were analyzed to assess coronal alignment and patellar engagement.

### 2.5. Data Analyses 

Statistical analysis was performed to evaluate the changes in PROMs and ROM before and after TKA. As the normality test was not satisfied due to the small sample size, the Wilcoxon signed-rank test was used for analysis and performed with the SPSS^®^ for Windows^®^ statistical software package (version 25, SPSS Inc., Chicago, IL, USA). A *p*-value of less than 0.05 was considered statistically significant.

## 3. Results

There were significant improvements observed in all PROMs and knee ROM values at the postoperative examination after two years (all *p* < 0.05) (Table 2). Only one case (Case No. 2) experienced a persistent extension lag, of 15°. Remarkably, the patient had adapted to this condition over an extended period and did not express significant discomfort or complaints.

The mean preoperative mechanical femorotibial angle was 8.2 ± 6.5° valgus, which changed to 0.7 ± 1.6° valgus at the last follow-up. The mean preoperative patellar lateral tilt angle was 25.8 ± 15.0°, indicating lateral dislocation, and improved to 2.8 ± 4.2° (Table 2). During the follow-up, no patients experienced significant complications such as infection, instability, loosening, dislocation, or implant malposition that required revision surgery. One patient showed patellar lateral subluxation on radiographs after 30 months post-surgery, but there was no dislocation, and the subluxation did not progress until the last follow-up at 41 months. Additionally, one patient reported subjective knee weakness, while another patient required the use of a cane for walking long distances.

## 4. Discussion

The most important finding of this study is the efficacy of primary TKA in achieving satisfactory survivorship and clinical outcomes for patients with neglected patellar dislocation. Accompanying procedures within the TKA spectrum, including lateral release, medial plication, and quadriceps lengthening, adequately addressed the patient-specific pathologies associated with neglected patellar dislocation.

The literature on the surgical treatment of knees with neglected dislocation of the patella and OA is lacking in number. Several case reports have been published, but they involved fewer than three patients, and there are inconsistencies in surgical techniques and clinical outcomes [6,7,8,10,11,12,13,14,15,16,17,18]. Therefore, it may be confusing to make treatment plans for these patients. Marmor successfully treated a patient with bilateral congenital patellar dislocation using TKA without repositioning the dislocated patella and extensor mechanism [10]. Four years post-surgery, the patient demonstrated satisfactory stability and quadriceps strength. Marmor’s approach suggests avoiding repair of the extensor mechanism in cases where patients have adapted to the disability [10]. Pradhan et al. also performed TKA on bilateral patellar dislocation with secondary OA without reconstructing the extensor mechanism, based on Marmor’s report [12]. However, the patient experienced knee dislocation 14 months after TKA in the right knee, necessitating two revision surgeries and the application of a constrained-type prosthesis. Based on this experience, they recommend the use of a constrained-type prosthesis in cases requiring extensive release [12]. Yamanaka et al. reported a case of patellar dislocation managed with TKA combined with distal realignment by tibial tubercle transfer. At the final follow-up two years after the operation, the patient achieved full extension, 90° flexion, and improved quadriceps strength, compared to before surgery [17]. Kubo et al. reported a lateral parapatellar approach for a patient with patellar dislocation. However, this approach is limited by its infrequent use among surgeons due to its relative unfamiliarity and poor visualization compared to the medial parapatellar approach [26]. While these prior studies introduced a range of surgical interventions, from soft tissue to additional bony surgeries, their applicability is limited due to the niche nature of neglected patellar dislocation, resulting in a lack of evidence for reproducibility. Our study showed that soft tissue procedures within the scope of TKA, coupled with the use of condylar constraint as required, are sufficient for the effective management of neglected patellar dislocation.

Valgus deformities are frequently encountered in cases of neglected patellar dislocation. These deformities can be corrected effectively through precise bone cutting and soft tissue balancing. If there is narrowing of the lateral extension gap following distal femur and proximal tibia resection, the rectangular extension gap can be restored through transverse stab-incision and the pie-crusting technique for the ITB, PLC, LCL, and popliteus tendon. The rotation angle of the femoral component required to establish a rectangular flexion gap was ascertained while the leg was maintained in a 90° knee flexion position, leveraging the gravitational tension of the gap induced by the weight of the patient’s lower leg [23]. 

Displacement of the extensor mechanism along with the contracture of the lateral knee structures occurs when the patella is dislocated. This leads the short quadriceps tendon to become permanently displaced laterally, exerting a lateral force on the tibia. Consequently, this results in an external rotation deformity of the tibia and lateralization of the tibial tubercle [27]. These deformities were addressed through tibial-component positioning in this study. In a previous study, ATCC was found to be the most reliable anatomical landmark for determining tibial rotational alignment in TKA [24]. Furthermore, by combining the ATCC method with the lateralization of the tibial component, the necessity for distal alignment surgery was eliminated, resulting in satisfactory outcomes in all examined cases.

To correct the patellofemoral maltracking caused by multiple factors associated with neglected patellar dislocation, surgical techniques to restore the quadriceps angle (Q-angle) are necessary. Lateralizing the femoral and tibial components while medially adjusting the patellar component offers the potential of reducing the Q-angle, enhancing the extensor mechanism, and correcting patellar hypoplasia [28,29]. This approach can also reduce tibial subluxation. In cases where the remaining patellar bone stock exceeds 12 mm, patellar resurfacing may yield congruence for patellofemoral tracking [25]. In addition, advancements in modern femoral component design enable the resolution of trochlear groove dysplasia, resulting in improved tracking that resembles normal patellofemoral kinematics [30].

In this study, extensive lateral release was performed in all cases following component implantation. This was due to inadequate patellofemoral tracking caused by a tight lateral and loose medial retinaculum. For cases No. 2 and No. 3, medial plication was added when lateral release was insufficient for correcting patellofemoral maltracking. 

**Figure 3:**
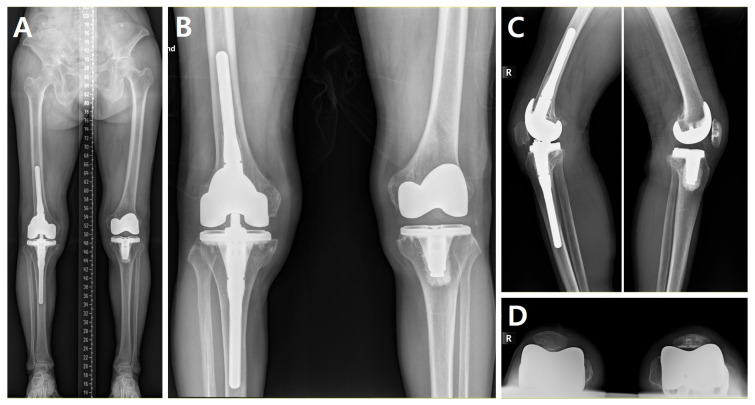
(**A**–**D**). Postoperative plain radiographs (Cases No. 1 and No. 2): (**A**) long-leg standing anteroposterior view; (**B**) standing anteroposterior view; (**C**) lateral view of right and left knees; and (**D**) skyline view.

To address the shortened quadriceps, we performed quadriceps lengthening procedures, specifically the quadriceps snip and V-Y quadricepsplasty. For cases No. 5 and No. 12, the quadriceps snip was considered more appropriate due to its relative ease of execution, reparability, and the advantage of providing a clear visualization of the surgical field. Meanwhile, Case No. 2 had an MFTA exceeding 20° and a TT-TG of 26.1, indicative of severe valgus and a significantly shortened quadriceps. This case also exhibited an intraoperative limited flexion of 60°. Consequently, we performed a lengthening procedure via V-Y quadricepsplasty for this case. This technique allows for extensive quadriceps lengthening and provides the ability to tension the quadriceps to the desired degree. While V-Y quadricepsplasty offers these advantages, it is more invasive than the quadriceps snip procedure. Hence, we confined its use to this particularly challenging case. A condylar constrained prosthesis was used to solve a significant flexion gap for this case (Figure 3), and no rotating hinge implants were used.

There are some limitations to this study. Firstly, the retrospective design precluded comparison with control groups and introduced potential selection bias. In addition, due to the rare nature of the condition, a sample-size calculation was not feasible, and the small sample size limits the statistical power of our analysis. There may also be a broader spectrum of pathologies not represented in our case series, and pathology-specific additional procedures could have further influenced outcomes. The collection and analysis of PROMs were limited to the 2-year postoperative period due to missed visits beyond this timeframe in certain cases, thereby impeding the extended statistical analysis of clinical outcomes. Nonetheless, radiological assessments were conducted until the last follow-up visit. Lastly, since the study was conducted in South Korea, its findings may have limited generalizability to other ethnic groups.

## 5. Conclusions

Primary TKA proved to be an effective surgery for correcting the various pathologies in neglected patellar dislocation with end-stage OA without necessitating additional bony procedures. The application of pathology-specific additional surgical techniques addressing the respective pathologies resulted in satisfactory clinical and radiological outcomes. 

## Figures and Tables

**Table 1:** Summary of demographics, preoperative associated problems, and additional procedures of the 12 cases in this study.

No.	Sex/Age	Side	Preoperative Associated Problems									Additional Procedures		
			MFTA (Valgus)	Subluxation of Tibia	External Torsion of Tibia	Lateralization of Tibial Tuberosity	Trochlear Dysplasia	TT-TG (mm)	Patella Hypoplasia/Thickness, (mm)	Retinacular Imbalance	Shortened Quadriceps	Lateral Release	Medial Plication	Quadriceps Procedures
1	F/68	Left	2.3	+	+		+	15.4	+/16	+		+		
2	F/68	Right	20.8	+	+	+	+	26.1	−/18	+	+	+	+	VY-Q
3	F/76	Left	10.1		+		+	15.6	−/22	+		+	+	
4	F/76	Right	10.2		+		+	12.3	−/16	+		+		
5	F/60	Left	10.4	+	+	+	+	27.1	−/11	+	+	+		Q-snip
6	M/66	Right	11.0		+			15.7	+/18	+		+		
7	F/64	Left	0.4		+		+	19.1	+/17	+		+		
8	F/64	Right	0.73		+	+	+	24.5	+/20	+		+		
9	F/71	Right	4.6		+	+	+	20.5	+/14	+		+		
10	F/71	Left	5.0		+		+	16.4	+/13	+		+		
11	F/70	Right	2.3	+	+	+	+	23.7	+/20	+		+		
12	M/57	Right	17.6	+	+	+		20.2	−/22	+	+	+		Q-snip

MFTA, mechanical femorotibial angle; TT-TG, tibial tuberosity–trochlear groove distance; Q-snip, quadriceps snip; VY-Q, VY quadricepsplasty.

**Table 2:** Clinical and radiological outcomes.

	Preoperative	Postoperative	*p*-Value
Knee Society Score			
Knee score	36.7 ± 16.5	88.3 ± 5.3	<0.01 *
Function score	35.0 ± 12.8	85.5 ± 7.8	<0.01 *
WOMAC	52.8 ± 7.7	14.3 ± 4.9	<0.01 *
ROM (°)	112.1 ± 17.8	125.0 ± 6.1	0.01 *
Flexion contracture	8.3 ± 10.7	1.7 ± 2.5	0.03 *
Flexion angle	120.4 ± 15.6	126.7 ± 6.8	0.07
Mechanical femorotibial angle (°)	8.2 ± 6.5 ^†^	0.7 ± 1.6 ^†^	0.02 *
Patellar lateral tilt angle (°)	25.8 ± 15.0	2.8 ± 4.2	<0.01 *

Results are presented as means ± standard deviations. Statistical analysis was performed using the Wilcoxon signed-rank test. Postoperative Knee Society Scores, WOMAC determinations, and ROM data were collected at 2 years. Postoperative mechanical femorotibial angle and patellar lateral tilt angle were collected at the last follow-up visit. WOMAC, Western Ontario and McMaster Universities Osteoarthritis Index; ROM, range of motion. *: statistically significant at *p* < 0.05. ^†^: valgus.

## Data Availability

The original contributions presented in the study are included in the article; further inquiries can be directed to the corresponding author/s.
